# Interaction Between *Enterococcus faecalis* and *Fusobacterium nucleatum* Regulated Macrophage Transcriptional Profiling and Reprogrammed Cellular Immune and Metabolic Response

**DOI:** 10.3390/microorganisms13061351

**Published:** 2025-06-11

**Authors:** Jingheng Liang, Wenling Huang, Poukei Chan, Lihong Guo

**Affiliations:** 1 Hospital of Stomatology, Sun Yat-sen University, Guangzhou 510055, China; 2 Guangdong Provincial Key Laboratory of Stomatology, Sun Yat-sen University, Guangzhou 510055, China

**Keywords:** *E. faecalis*, *F. nucleatum*, bacterial interaction, macrophage, refractory apical periodontitis

## Abstract

Refractory apical periodontitis (RAP), a persistent infection after root canal treatment, still has no effective treatment. *Enterococcus faecalis* (*E. faecalis*) and *Fusobacterium nucleatum* (*F. nucleatum*) are frequently detected in the lesion. We previously found that coaggregation altered gene expression of *E. faecalis* and *F. nucleatum* and promoted immune evasion by suppressing pro-inflammatory cytokine secretion of macrophages (Mφs) while sustaining low-grade inflammation. In this study, we further investigated the synergistic effect of coaggregated *E. faecalis* and *F. nucleatum* on modulating Mφ immune and metabolic responses. Using transmission electron microscope, flow cytometry, RNA-seq and functional assays, we demonstrated that coaggregated *E. faecalis* and *F. nucleatum* caused nuclear shrinkage and increased mitochondria in Mφ while inducing M1 polarization, ROS production, and lipid accumulation of Mφ. The key driver genes causing the difference between single species-infected and coaggregated bacteria-infected Mφ mainly included IFN-stimulated genes and genes related to the chemokine signaling pathway. These findings indicate that the synergism of *E. faecalis* and *F. nucleatum* can regulate the immune and metabolic response of Mφ, offering novel insights into therapeutic targets for refractory apical periodontitis.

## 1. Introduction

Chronic apical periodontitis is inflammatory disease caused by persistent microbial infection or pathogenic stimuli within the root canal. The global prevalence of apical periodontitis reaches up to 52%, making it a significant factor in tooth loss [1]. Moreover, apical periodontitis may be an important risk factor for systemic diseases such as cardiovascular diseases, diabetes, and adverse pregnancy outcomes [2].

However, conventional root canal treatment cannot completely cure all cases of chronic apical periodontitis, leading to the occurrence of refractory apical periodontitis (RAP), characterized by persistent inflammation and progressive alveolar bone destruction [3]. Revealing the pathological mechanisms of refractory apical periodontitis and developing effective prevention and treatment strategies are of great significance.

Macrophages (Mφs) can release pro-inflammatory cytokines through necroptosis and regulate the inflammatory response through polarization in refractory apical periodontitis [4]. In this condition, the proportion of the pro-inflammatory M1-type Mφ increases, accompanied by increased expression of IL-6 and the production of reactive oxygen species (ROS). These changes can lead to sustained inflammation and may further mediate the destruction of bone tissue [5]. Additionally, some pathogens can persist within Mφs while inducing low-grade inflammatory responses, resulting in the persistence of infection and inflammation [6,7]. Therefore, the interaction between pathogens and Mφs plays an important role in the process of refractory apical periodontitis.

Refractory apical periodontitis is often accompanied by high detection rates of two important pathogenic bacteria, *Enterococcus faecalis* (*E. faecalis*) and *Fusobacterium nucleatum* (*F. nucleatum*), with up to 77% and 64%, respectively [4,8], Both *E. faecalis* and *F. nucleatum* possess multiple virulence factors, strong adhesion capabilities, and the ability to evade host immune clearance [6,9]. *E. faecalis* can survive in dentinal tubules and resist the sodium hypochlorite irrigation in conventional root canal treatment while surviving within Mφ for up to 72 h, inducing the production of pro-inflammatory cytokines such as TNF-α and leading to the persistence of infection [6,10]. *F. nucleatum* plays an important role in the synergistic pathogenicity of the bacterial community, as it has multiple adhesins. It can also interfere with the maturation of phagosomes, allowing bacteria to persist in Mφs [9,11].

The microbial community is not just a physical combination of all bacteria but is composed of bacterial species that have specific interactions. *F. nucleatum* can reduce the redox potential, thereby improving the growth conditions for *E. faecalis*, suggesting a possible synergistic interaction between the two bacteria [12]. According to the literature, compared to single-species infections, mixed infections of *E. faecalis* and *F. nucleatum* in the root canal system of mice result in high expression of different inflammatory cytokines in the periapical tissues, and the expression of chemokines and their receptors is significantly upregulated [12,13].

Coaggregation is a process of mutual recognition between bacterial species, produced by the interaction of lectin-like substances and carbohydrates, leading to physical adhesion, and it is considered key to promoting interactions between bacteria [14]. Approximately 1000 bacterial species in the oral cavity have been confirmed to have at least one coaggregation partner, with high specificity [14].

We previously found that *F. nucleatum* could firmly coaggregate with *E. faecalis*, which improved *E. faecalis*’s tolerance to stress environments such as strong alkali, high salt, oligotrophic conditions, antibiotics, and chlorhexidine, leading to a marked improvement in bacterial survival rate [15]. Additionally, after coaggregation, the transcription levels of a series of genes in both bacteria changed significantly [15]. This suggests that the physical coaggregation of *E. faecalis* and *F. nucleatum* promotes the evolution of their physiological functions, allowing both to gain evolutionary and survival advantages through the synergistic action of the microbial community. More importantly, this advantage may significantly enhance the ability of pathogenic bacteria to evade host immune clearance and cause further disease.

Indeed, our preliminary findings show that after infection with coaggregated *E. faecalis* and *F. nucleatum*, the enrichment and survival ability of *E. faecalis* within the Mφ significantly increased. The Mφ produced more pro-inflammatory cytokines and chemokines compared to the blank group but less compared to infection with non-coaggregated bacteria [15]. This suggests that coaggregation of *E. faecalis* and *F. nucleatum* facilitates their evasion of host immune clearance, providing opportunities for their spread within the host and promoting a low-grade inflammatory response in Mφs, leading to persistent inflammation. However, the mechanisms behind this need further exploration.

Therefore, this study aimed to further investigate the regulatory effects of coaggregated *E. faecalis* and *F. nucleatum* on Mφs ultrastructural changes, polarization, ROS production, and lipid metabolism while comparing the impact of single-species and coaggregated bacteria on the Mφ transcriptome so as to provide new insights into the mechanisms by which the two bacteria synergistically regulate and evade host immunity. The findings of this study may offer novel insights into therapeutic targets for refractory apical periodontitis.

## 2. Materials and Methods

### 2.1. Bacterial Strains and Culture Conditions

*E. faecalis* OG1RF ATCC 47,077 and *F. nucleatum* subsp. *polymorphum* ATCC 10,953 were acquired from Guangdong Microbial Culture Collection Center (GDMCC; Guangzhou, China). *E. faecalis* was cultured in Brain Heart Infusion (BHI) broth (Difco, Franklin Lakes, NJ, USA) aerobically, and *F. nucleatum* was cultured in BHI broth containing 5 g/L yeast extract (Thermo Fisher Scientific, Waltham, MA, USA), 0.4 g/L L-cysteine HCL (Amresco, Solon, OH, USA), 0.005 g/L hemin (Solarbio, Beijing, China), and 0.001 g/L vitamin K (Ronshyn, Shanghai, China) anaerobically (5% CO_2_, 5% H_2_, 90% N_2_).

### 2.2. Culture and Differentiation of THP-1 Cells

The human monocyte cell line THP-1 was kindly provided by Stem Cell Bank, Chinese Academy of Sciences (Shanghai, China). THP-1 monocytes were grown in RPMI 1640 supplemented with 10% fetal bovine serum (FBS) (Australian origin, Thermo Fisher Scientific, MA, USA) in a CO_2_ incubator (5% CO_2_ humidified atmosphere at 37 °C). Cells were treated with 50 ng/mL phorbol 12-myristate 13-acetate (PMA, Macklin Inc., Shanghai, China) for 48 h for Mφ differentiation. The THP-1-derived Mφ (dTHP-1 cells) were then grown in fresh RPMI 1640 supplemented with 10% FBS for 24 h in a CO_2_ incubator.

### 2.3. Establishment of Coaggregation Model

Coaggregation buffer (CAB) (150 mM NaCl, 1 mM Tris pH 8.0, 0.1 mM CaCl_2_, and 0.1 mM MgCl_2_) was prepared as previously described [16]. The intergeneric coaggregation model of *E. faecalis* and *F. nucleatum* was established as previously described with minor modifications [15]. Briefly, *E. faecalis* and *F. nucleatum* at the late logarithmic growth phase were, respectively, washed twice and resuspended in CAB and adjusted to a final concentration of 2 × 10^9^ CFU/mL. *E. faecalis* and *F. nucleatum* were allowed to coaggregate by mixing equal suspensions of bacterial cells in a reaction tube, vortexed for 20 s, and left undisturbed anaerobically at room temperature for 10 min. After incubation, coaggregated *E. faecalis* and *F. nucleatum* were pelleted by low-speed (200× *g*) centrifugation for 1 min, while non-coaggregated bacteria were dispersed in the supernatant; then, the supernatant was collected carefully and measured for OD_600nm_ (OD*_Ef-Fn_*).

The degree of coaggregation was evaluated by the coaggregation index (CI) calculated according to the following formula: $CI=\frac{{OD}_{Ef}+{OD}_{Fn}-{OD}_{Ef-Fn}}{{OD}_{Ef}+{OD}_{Fn}}\times 100\%$ [17,18]. OD*_Ef_* and OD*_Fn_* represents the optical density of *E. faecalis* and *F. nucleatum* at 600 nm, respectively. The coaggregated bacteria with CI over 70% were collected for further experiments [15].

### 2.4. Co-Culture of dTHP-1 Cells and Bacterial Cells

The bacterial invasion assay was performed as described previously with some modifications [19]. dTHP-1 cells were seeded in sterile Corning™ Costar™ flat-bottom 6-well cell culture plates (1.0 × 10^6^ cells/well) and incubated for 12 h before use. dTHP-1 cells were infected with *E. faecalis*, *F. nucleatum*, or coaggregates of the two species, respectively (multiplicity of infection (MOI) was 10:1).

We conducted a preliminary test to determine the bacterial density of the coaggregates before treating the dTHP-1 cells [19]. In detail, the supernatant of the coaggregation group containing non-aggregating cells was removed. The pellets (coaggregates) were resuspended in PBS, and the tube was vigorously vortexed until no pellet was visible. Then, the suspension was diluted and seeded onto sheep blood agar plates as well as BHI agar plates so as to determine the bacterial density. After the preliminary test, the bacterial volumes of the monoculture and coaggregates were adjusted to reach a similar bacterial density and met the MOI (10:1) requirement.

After incubation of dTHP-1 cells in a CO_2_ incubator for 2 h, the plates were washed three times with PBS and cultured in fresh RPMI 1640 containing 10% FBS, gentamicin (300 mg/mL), and metronidazole (200 mg/mL) (Solarbio, Beijing, China) to kill extracellular bacteria. After 2 h, the plates were washed three times with PBS and cultured in fresh RPMI 1640 containing 10% FBS for 6 h before further experiments.

### 2.5. Transmission Electron Microscopy (TEM) Imaging

TEM was used to visualize *E. faecalis* and *F. nucleatum* within the dTHP-1 cells as well as changes in organelles of Mφs. The infected dTHP-1 cells were fixed with 2.5% glutaraldehyde (Sinopharm, Beijng, China) overnight. After washing twice with PBS, the cells were postfixed with 1% OsO_4_ (SPI-CHEM, West Chester, PA, USA) in phosphate buffer (0.1 M, pH 7.0) for 1~2 h and then washed twice with PBS again. After fixation, samples were dehydrated with gradient ethanol (Sinopharm, Beijing, China), transferred to absolute acetone (Sinopharm, Beijing, China), and infiltrated with a Spurr resin (SPI-CHEM, PA, USA) mixture. The embedded samples were sectioned in a microtome (LEICA EM UC7, Wetzlar, Germany), stained with uranyl acetate (SPI-CHEM, PA, USA) and lead citrate (SPI-CHEM, PA, USA), and viewed on a Hitachi Model H-7650 TEM (Hitachi, Tokyo, Japan).

### 2.6. Fluorescence Flow Cytometric Analysis (FACS) of Cell Surface Markers

After 2 h of incubation and 2 h of antibiotic killing of extracellular bacteria, dTHP-1 cells were further incubated for 2, 6, and 24 h, respectively. Afterwards, the cells were harvested for phenotypic identification of Mφ. After washing with ice-cold FACS buffer (1% BSA–PBS), cells were stained with APC-labeled anti-CD80 (Biolegend, San Diego, CA, USA) and PE-labeled anti-CD206 (Biolegend, San Diego, CA, USA) at 4 °C for 40 min. Then, FACS buffer washes were performed to remove excess antibody, and the samples were subjected to detection and analysis using a FACSCalibur flow cytometer (Cytoflex, Beckman, Brea, CA, USA) along with FlowJo software v10.

### 2.7. ROS Determination

The production of ROS was measured using the oxidation-sensitive probe, 2′,7′-dichlorofluorescein diacetate (DCFH-DA, Applygen, Beijing, China). Briefly, after 2 h of incubation and 2 h of antibiotic killing of extracellular bacteria, dTHP-1 cells were further incubated for 6 h; then, the cell supernatant was discarded. Serum-free medium was added and pipetted to dislodge the attached cells. The cell pellets were collected and washed twice with serum-free medium before staining with DCFH-DA at a final concentration of 10 μM at 37 °C in the dark for 40 min. The cells were then washed twice with cold PBS and resuspended in PBS for analysis of intracellular ROS by a FACSCalibur flow cytometer (Cytoflex, Beckman, Brea, CA, USA) along with FlowJo software. Additionally, the fluorescence intensity was detected at an excitation wavelength of 502 nm and an emission wavelength of 530 nm using a fluorescence plate reader (Biotek, Winooski, VT, USA), and the fluorescence was observed with a fluorescence microscope (Zeiss, Oberkochen, Germany). Infected cells treated with H_2_O_2_ at 48 μM served as a positive control, and cells not infected by bacteria served as a negative control.

### 2.8. RNA Extraction

The total RNA of the dTHP-1 cells infected with coaggregated pellets or monocultures of *E. faecalis* and *F. nucleatum* was extracted using RNAzol^®^RT according to the manufacturer’s instructions (MRC, Cincinnati, OH, USA). The extracted RNA was dissolved in 30 μL RNase-free water. RNA was quantified using NanoDrop-2000 (NanoDrop Technologies, Wilmington, DE, USA).

### 2.9. RNA Sequencing

RNA sequencing was conducted using the DNBSEQ platform. SOAPnuke (v1.6.5) was applied for filtering reads and obtaining clean reads; reads with adapters, more than 1% unknown bases, and low quality were removed. The clean reads were mapped to the human reference genome GRCh38.p12 using HISAT2 V2.0.4. Gene expression levels were calculated using Bowtie2 V2.4.5 and RSEM V1.3.1. Stringent criteria, including Log_2_FC > 1 and false-discovery rate (FDR < 0.001), were applied to filter differentially expressed genes (DEGs).

Data mining and figure presentation, including Kyoto Encyclopedia of Genes and Genomes (KEGG) classification, enrichment and network analyses, Gene Ontology (GO) classification analysis, cluster heatmap, principal component analysis (PCA), protein–protein interaction (PPI) analysis, and key driver analyses (KDA) of DEGs, were all performed using the BGI in-house customized data mining system called Dr.Tom (http://report.bgi.com accessed on 16 April 2025). RNA sequencing data supporting the findings of this study were deposited in the NCBI Sequence Read Archive (SRA) under accession number PRJNA1243572.

### 2.10. Quantitative Reverse Transcription PCR (qRT-PCR)

qRT-PCR was used to validate changes in mRNA expression of DEGs. The total RNA of dTHP-1 cells infected with coaggregated pellets or monocultures of *E. faecalis* and *F. nucleatum* was extracted and examined as previously described. RNA reverse transcription was performed according to the manufacturer’s instructions (PrimeScript™ RT Master Mix (Perfect Real Time), TAKARA, Shiga, Tokyo, Japan). Subsequent quantitative real-time PCR amplification was performed to detect relative mRNA expression levels using SYBR^®^ Premix Ex Taq™ II (Tli RNaseH Plus) (TAKARA, Shiga, Tokyo, Japan) following the manufacturer’s protocol on a LightCycler 96 system (Roche, Basel, Switzerland). The 2^−ΔΔCt^ method was used to quantify fold induction by normalizing to the reference gene, *GAPDH*. Gene-specific primer sequences are listed in Table S1.

### 2.11. Oil Red O Staining

Oil Red O staining was used to measure lipid accumulation within the Mφ. Briefly, 4% paraformaldehyde-fixed cells were treated with 60% isopropanol for 5 min, followed by incubation with Oil Red O solution (OriCell, Guangzhou, China) for 40 min. After washing twice with PBS, the cells were counter-stained with hematoxylin for 30 s. Finally, the stained cells were observed under an inverted microscope. For quantification, after incubation with Oil Red O solution, the excess dye solution was discarded, and the cells were washed twice with PBS followed by dissolution in 100% isopropanol for 30 min. Optical densities were measured at 500 nm using a microplate reader.

### 2.12. Statistical Analysis

Statistical analyses and illustrations were performed with GraphPad Prism (version 10.1.2). In addition to transcriptomics analysis, the significance of statistical differences (*p* < 0.05) was determined through one-way analysis of variance (ANOVA) or unpaired *t*-test. All experiments were performed in triplicate.

## 3. Results

### 3.1. Coaggregated E. faecalis and F. nucleatum Cause Nuclear Shrinkage and Increased Mitochondria in Mφ

**Figure 1:**
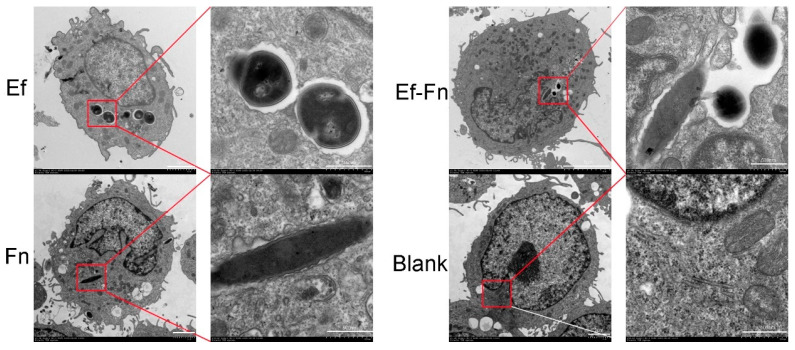
Transmission electron microscopy (TEM) images of dTHP-1 cells infected with a single species or coaggregated *E. faecalis* and *F. nucleatum*. (Ef: *E. faecalis*; Fn: *F. nucleatum*; Ef-Fn: coaggregated *E. faecalis* and *F. nucleatum*).

Through TEM, it was observed that the invaded bacteria in the Mφ were located within phagosomes. Specifically, coaggregated *E. faecalis* and *F. nucleatum* were found in the same phagosome, with extracellular matrix connections between them. In addition, even after being phagocytosed, *E. faecalis* was still observed in a dividing state. It is worth noting that compared with the negative control group, Mφs infected with *F. nucleatum* or coaggregated *E. faecalis* and *F. nucleatum* showed significant nuclear shrinkage, while those infected with *E. faecalis* had relatively intact nuclear morphology. Moreover, Mφs infected with coaggregated *E. faecalis* and *F. nucleatum* exhibited a significant increase in mitochondria, suggesting that coaggregated bacteria may regulate the metabolism of Mφs in immune response (Figure 1).

### 3.2. Coaggregated E. faecalis and F. nucleatum Induce M1 Polarization of Mφ

**Figure 2:**
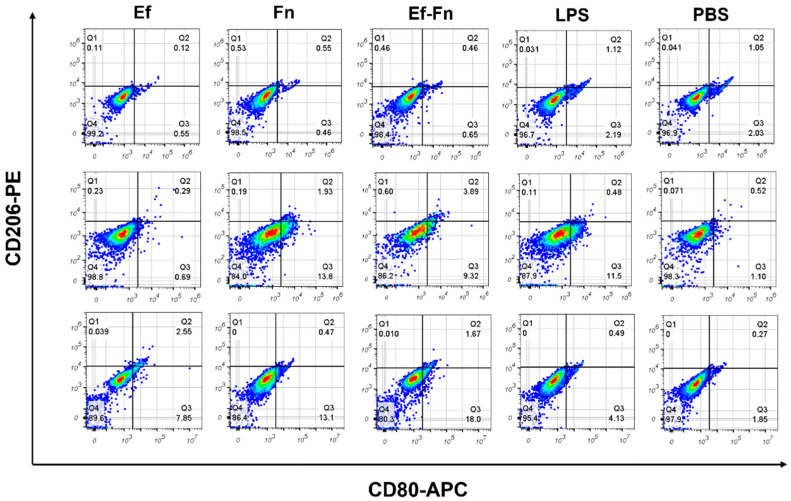
Flow cytometric analysis of CD80-APC and CD206-PE staining of Mφs treated with a single species or coaggregated bacteria or LPS (100 ng/mL) for 2, 6, and 24 h.

In flow cytometry, cells expressing CD80^+^CD206^−^ are considered of the M1-like phenotype Mφ, whereas CD80^+^CD206^+^-expressing cells are considered of the M2-like phenotype Mφ. The results showed that 2 h after bacterial invasion, there was no significant polarization tendency in any group of Mφ. However, at 6 h post invasion, cells in the *F. nucleatum* group and the coaggregated *E. faecalis* and *F. nucleatum* group exhibited significant M1 polarization, with the number of polarized cells in the coaggregated group slightly less than in the *F. nucleatum* group, while the *E. faecalis* group still showed no obvious polarization. At 24 h post invasion, the number of M1-polarized cells in the *F. nucleatum* group and the coaggregated group further increased, with the coaggregated group having more M1 Mφs than the *F. nucleatum* group, and the *E. faecalis* group also showed some M1-polarized Mφs (Figure 2). In summary, coaggregated *E. faecalis* and *F. nucleatum* can promote M1 polarization of Mφs, with this effect becoming more pronounced 6 h after bacterial invasion. Therefore, we used this time point for further experiments.

### 3.3. Coaggregated E. faecalis and F. nucleatum Promote Low-Level ROS Production in Mφ

ROS plays a central role in the phagocytosis of pathogens by Mφ, directly killing pathogens through oxidative mechanisms and enhancing antibacterial effects by increasing the expression of TNF-α, IL-1β, etc. Studies have shown that under inflammatory conditions (such as M1 polarization), Mφs can enhance the pentose phosphate pathway to promote ROS production [20].

**Figure 3:**
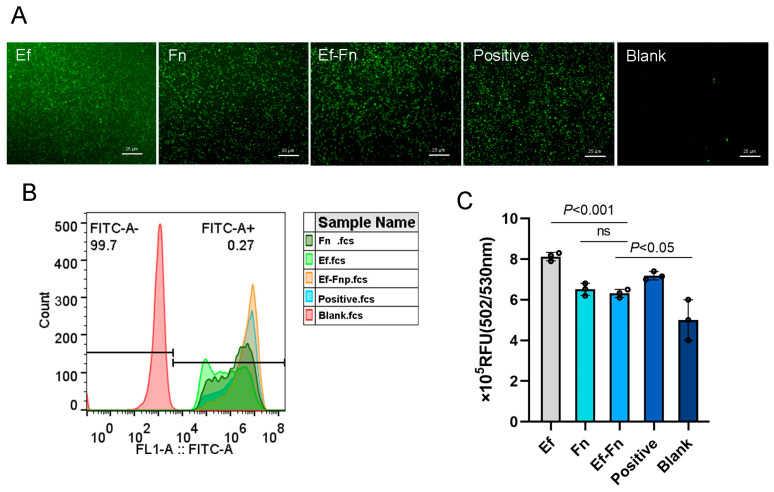
Detection of reactive oxygen species (ROS) production with fluorescent probe DCFH-DA. (**A**) Fluorescence microscope images. Stained ROS appeared green under a fluorescence microscope. Scale bar = 25 μm. (**B**) Flow cytometric analysis. (**C**) Optical density assays by fluorescence plate reader. Relative fluorescence unit (RFU) indicate fluorescence intensity. The larger the RFU value, the higher the detected fluorescence intensity.

We used the fluorescent probe DCFH-DA to detect ROS production before and after cell infection. Stained ROS appeared green under a fluorescence microscope. The results showed that coaggregated *E. faecalis* and *F. nucleatum* could induce ROS production in dTHP-1 cells, with the amount produced being less than in the *E. faecalis* group but similar to the *F. nucleatum* group (Figure 3), suggesting that coaggregated *E. faecalis* and *F. nucleatum* can promote low-level ROS production in Mφ.

### 3.4. Influence of E. faecalis and F. nucleatum Coaggregation on the Transcriptome of Mφ

**Figure 4:**
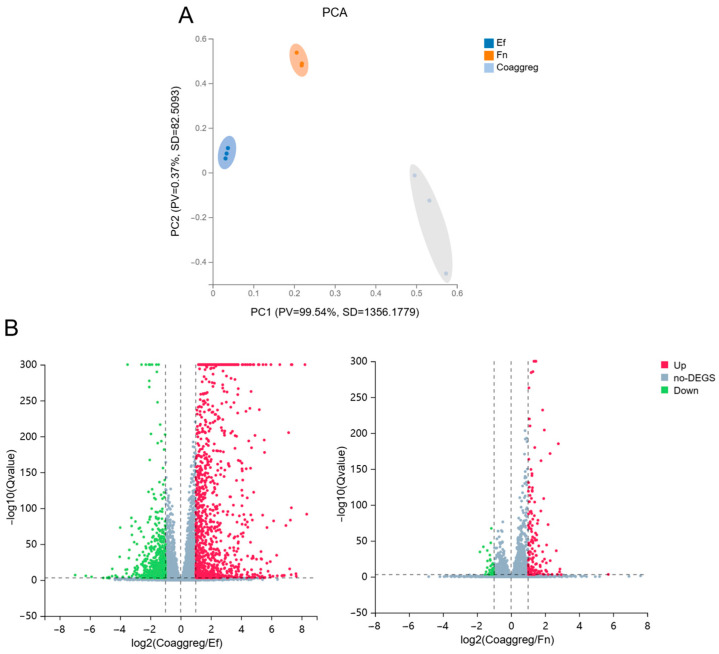
(**A**) Principal component analysis (PCA) of the Mφ transcriptome from three different treatments. (**B**) Volcano plot of the differentially expressed genes (DEGs) in Mφs infected with coaggregated bacteria vs. *E. faecalis* or *F. nucleatum*. The red and green spots denote upregulated genes and downregulated genes. The grey spots denote genes with no significant difference between Mφs infected with a single species or coaggregated bacteria. (Coaggreg: coaggregated *E. faecalis* and *F. nucleatum*; Ef: *E. faecalis*; Fn: *F. nucleatum*).

PCA analysis of the transcripts showed a clear separation between Mφ infected with a single species and with coaggregated bacteria, revealing coaggregation of *E. faecalis* and *F. nucleatum* as a main source of variance in the dataset (Figure 4A).

There were numerous and significant DEGs in Mφs infected with a single species versus coaggregated bacteria. In total, Mφs infected with coaggregated bacteria differentially expressed 1162 genes compared to those infected with *E. faecalis*, with 792 genes upregulated and 370 genes downregulated. Compared to Mφs infected with *F. nucleatum*, coaggregated bacterial infection led to differential expression of 124 genes, with 103 genes upregulated and 21 genes downregulated (Figure 4B and Figure S1). Details of the expression matrix and DEGs are provided in Tables S2 and S3.

#### 3.4.1. Coaggregated *E. faecalis* and *F. nucleatum* vs. *E. faecalis*

**Figure 5:**
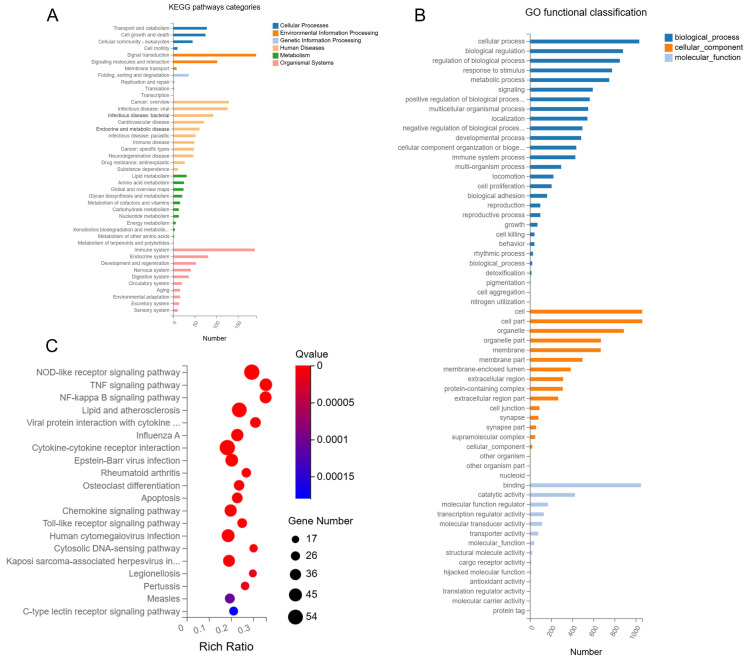
(**A**) Kyoto Encyclopedia of Genes and Genomes (KEGG) pathways categories. (**B**) Gene Ontology (GO) functional classification. (**C**) Bubble plots of KEGG pathway enrichment analysis.

KEGG pathway analysis showed that DEGs in Mφs infected with coaggregated bacteria were enriched in multiple pathways, including cellular processes, environmental information processing, genetic information processing, human diseases, metabolism, and organismal systems. Among these pathways, a significantly higher number of DEGs were involved in pathways of human diseases and organismal systems (Figure 5A).

Additionally, GO analysis revealed that DEGs in Mφ infected with coaggregated bacteria were enriched in biological processes, cellular components, and molecular functions, with biological processes occupying the most DEGs (Figure 5B).

In KEGG pathway enrichment bubble plots, the top 20 enriched pathways were ranked by FDR, including NOD-like receptor signaling pathway, TNF signaling pathway, NF-kappa B signaling pathway, lipid and atherosclerosis, and viral protein interaction with cytokine and cytokine receptor, most of which are directly related to Mφ inflammatory responses (Figure 5C). Among these, the NOD-like receptor signaling pathway and cytokine–cytokine receptor interaction pathways had the most DEGs, with 54 each.

Interestingly, among the top five enriched pathways, except for lipid and atherosclerosis, the others are directly related to inflammatory responses, suggesting that lipid metabolism may also be involved in the immune regulation of the infected Mφ. Therefore, we planned to verify the expression of key genes related to lipid metabolism and to detect the lipid accumulation of infected Mφs in the later study.

**Figure 6:**
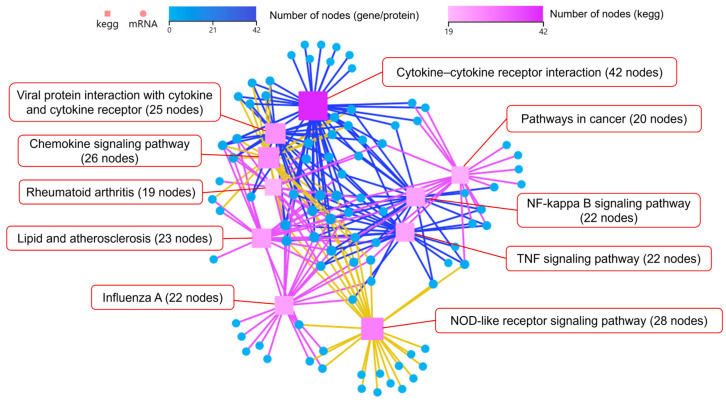
KEGG pathways network interaction analysis of DEGs in Mφs infected with coaggregated bacteria compared with *E. faecalis*.

Additionally, KEGG pathways network interaction analysis was performed to find out the connection among the top 10 pathways with the highest number of DEGs (Log_2_ fold-change (Log_2_FC) > 2). The results showed that DEGs mainly enriched in cytokine–cytokine receptor interaction, chemokine signaling pathway, and NOD-like receptor signaling pathway. The cytokine–cytokine receptor interaction pathway had the most node connections, indicating it has a profound impact on other enriched pathways (Figure 6).

**Figure 7:**
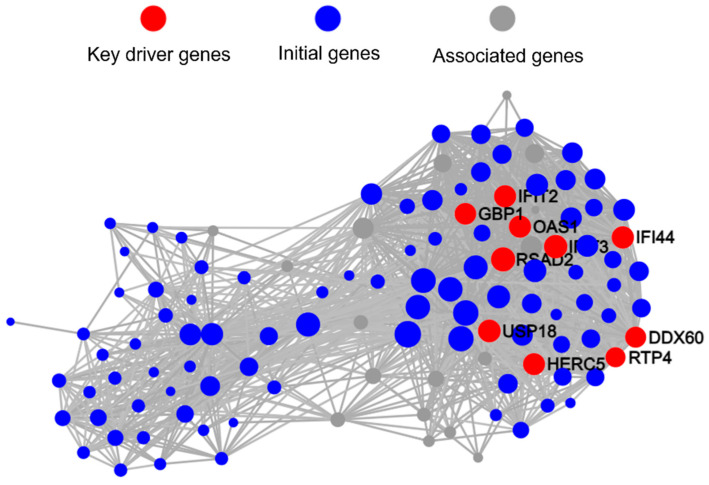
Key driver genes identified from the protein–protein interaction (PPI) network by key driver analyses (KDA).

We used PPI analysis to further analyze the network interactions of key DEGs. Interestingly, PPI analysis revealed that genes with Log_2_FC > 2 formed two main related clusters (Figure S2). Based on PPI analysis results, we conducted KDA to identify key driver genes and initial genes (Figure 7). The results showed that the top 10 key driver genes with the most node connections were all located in one cluster, and among them, only *OAS1* (54 nodes), *GBP1* (51 nodes), and *RSAD2* (66 nodes) had KEGG pathway annotations. These three genes were upregulated by 8.6-fold, 28.6-fold, and 35.3-fold, respectively, in the coaggregated bacteria group. *OAS1*, *GBP1*, and *RSAD2* are all interferon-stimulated genes (ISGs), with *OAS1* and *GBP1* being related to the NOD-like receptor signaling pathway.

#### 3.4.2. Coaggregated *E. faecalis* and *F. nucleatum* vs. *F. nucleatum*

**Figure 8:**
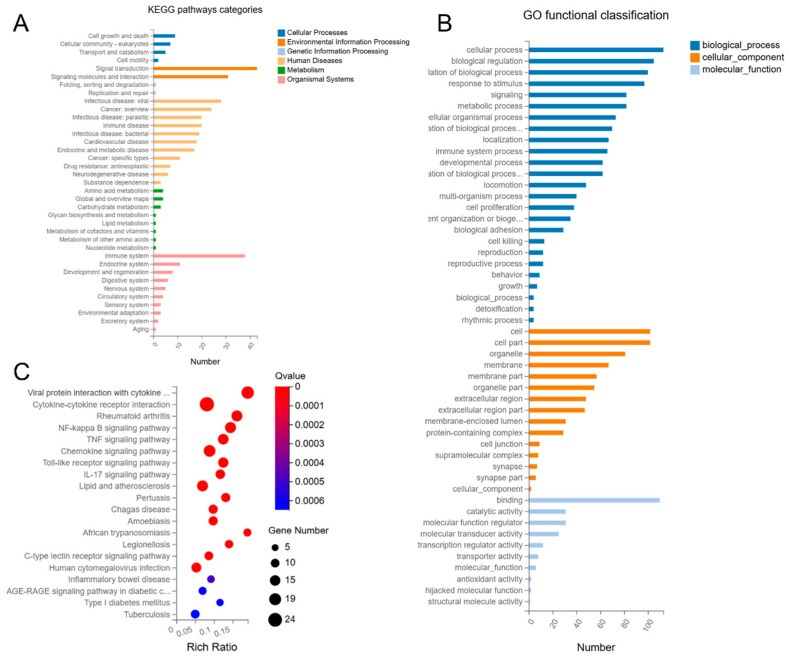
(**A**) KEGG pathways categories. (**B**) GO functional classification. (**C**) Bubble plots of KEGG pathway enrichment analysis.

KEGG pathway analysis showed that DEGs in Mφs infected with coaggregated bacteria were enriched in multiple pathways, including cellular processes, environmental information processing, genetic information processing, human diseases, metabolism, and organismal systems. Among these pathways, a significantly higher number of DEGs were involved in pathways of human diseases and organismal systems (Figure 8A).

Additionally, GO analysis revealed that DEGs were enriched in biological processes, cellular components, and molecular functions, with biological processes having the most differentially expressed genes (Figure 8B).

In KEGG pathway enrichment bubble plots, the top 20 enriched pathways include viral protein interaction with cytokine and cytokine receptor, cytokine–cytokine receptor interaction, rheumatoid arthritis, NF-kappa B signaling pathway, and TNF signaling pathway, etc. Among these pathways, some are related to Mφ inflammatory responses, while others are associated with parasitic, viral, and intracellular bacterial infections, suggesting that coaggregated *E. faecalis* and *F. nucleatum* may enhance their ability to survive as intracellular bacteria and activate Mφ immune responses (Figure 8C).

Among the top 20 enriched pathways, the cytokine–cytokine receptor interaction and viral protein interaction with cytokine and cytokine receptor pathways had the most DEGs, with 24 and 19, respectively. Interestingly, among the top five enriched pathways, except for rheumatoid arthritis, the others are related to inflammatory responses associated with bacterial infections, suggesting that, similar to the pathogenesis of rheumatoid arthritis, coaggregated *E. faecalis* and *F. nucleatum* may also induce sterile inflammatory responses in Mφs through cascade effects.

**Figure 9:**
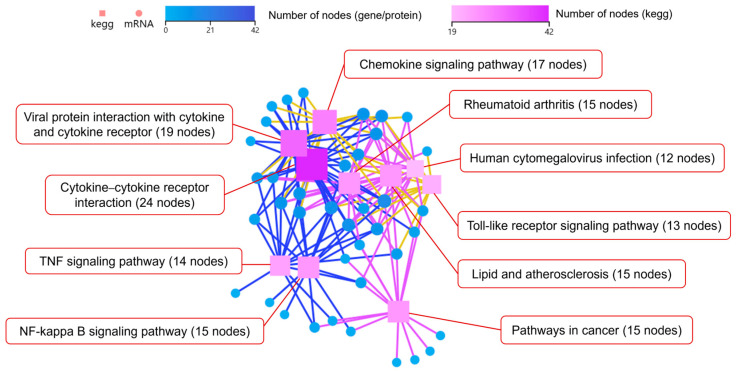
KEGG pathways network interaction analysis of DEGs in Mφs infected with coaggregated bacteria compared with *F. nucleatum*.

KEGG pathways network interaction analysis on the top 10 pathways with the highest number of DEGs showed that DEGs were mainly enriched in cytokine–cytokine receptor interaction, viral protein interaction with cytokine and cytokine receptor, and chemokine signaling pathway, with the cytokine–cytokine receptor interaction pathway having the most node connections, indicating its important influence on other enriched pathways (Figure 9).

**Figure 10:**
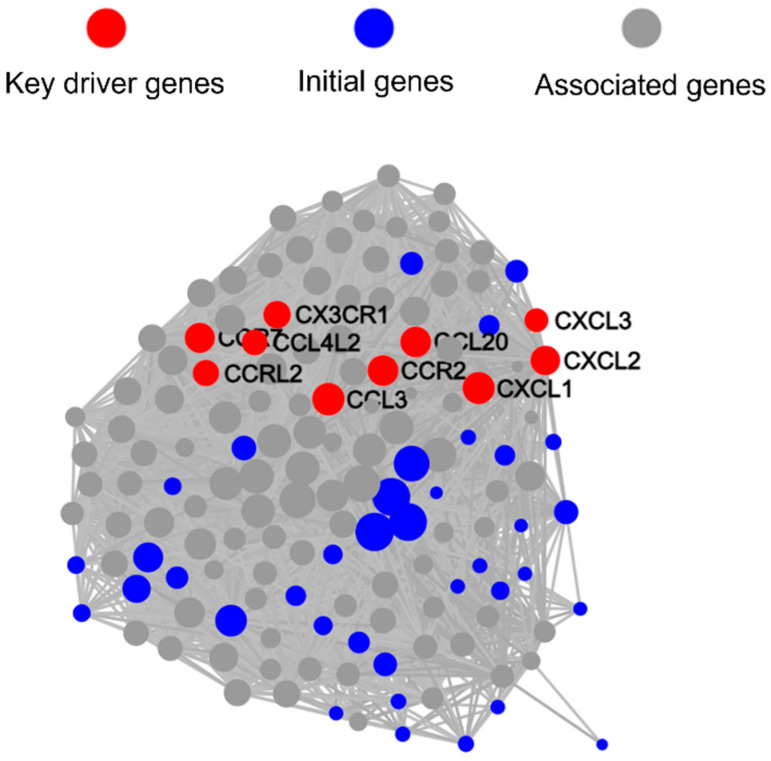
Key driver genes identified from the PPI network by KDA.

We used PPI to further analyze the network interactions of key DEGs, and the results showed that differentially expressed genes formed a single cluster (Figure S3). Based on PPI results, we conducted KDA, which revealed that the top 10 key driver genes with the most node connections were *CCL20* (83 nodes), *CXCL2* (77 nodes), *CXCL1* (92 nodes), *CXCL3* (46 nodes), *CCL4L2* (55 nodes), *CX3CR1* (65 nodes), *CCR2* (84 nodes), *CCRL2* (58 nodes), *CCR7* (80 nodes), and *CCL3* (96 nodes). Notably, all 10 key driver genes are related to the chemokine signaling pathway, and except for *CX3CR1* and *CCR2*, the other eight genes were upregulated, with fold changes ranging from 2.0 to 4.5 (Figure 10).

### 3.5. qRT-PCR Validation of Key DEGs Expression

In the RNA-seq results, we found that compared with *E. faecalis* single species, coaggregated *E. faecalis* and *F. nucleatum* could regulate the expression of key driver genes including *OAS1* (upregulated by 8.6-fold), *GBP1* (upregulated by 28.6-fold) and *RSAD2* (upregulated by 35.3-fold). Also, the apoptosis-related gens *Fas* was upregulated by 3.6-fold, which is consistent with the phenomenon of nuclear shrinkage observed by TEM. Moreover, it is interesting that DEGs were not only related to inflammatory responses but also significantly enriched in lipid metabolism-related pathways, with *SRC* gene expression upregulated by 18.1-fold.

In addition, compared with *F. nucleatum* single species, key driver genes regulated by coaggregated *E. faecalis* and *F. nucleatum* were all related to chemokine signaling pathway, including *CXCL1*, *CXCL2,* and *CXCL3*, which attract neutrophils through the same receptor, *CXCR2*. *CXCL3* expression was upregulated 2.4-fold.

**Figure 11:**
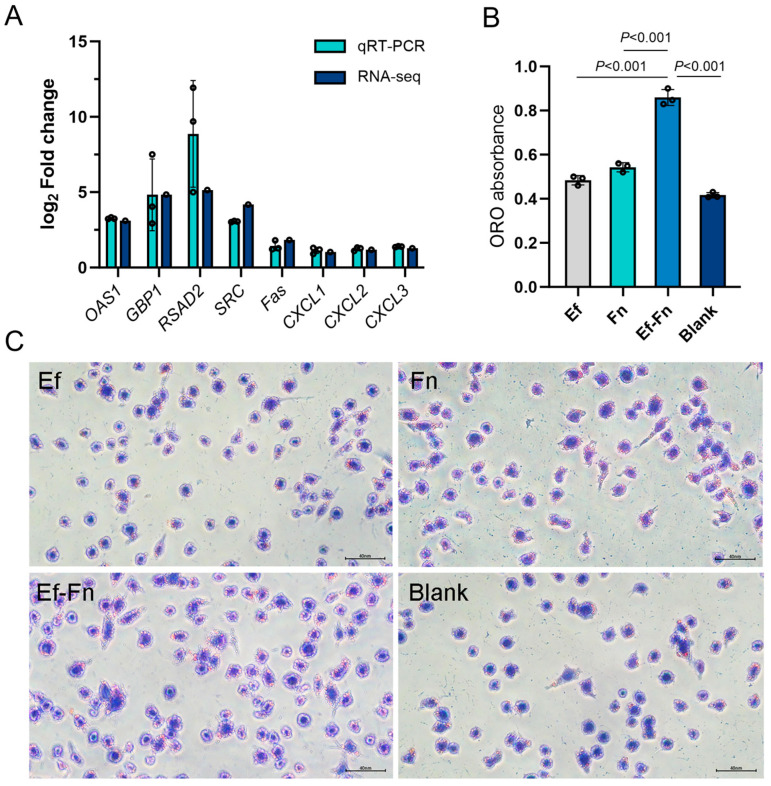
(**A**) qRT-PCR validation of DEGs expression in RNA-seq. (**B**) Optical density assay of Oil Red O-stained Mφ. (**C**) Representative images of Oil Red O-stained Mφ. Scale bar = 40 nm.

Therefore, we validated the expression of the aforementioned genes using qRT PCR. We found that the patterns of gene expression by RNA-seq and qRT-PCR were similar. There was a strong correlation between the two datasets, with Pearson’s correlation coefficients of 0.80 (Figure 11A).

### 3.6. Coaggregated E. faecalis and F. nucleatum Promote Lipid Accumulation in Mφ

The enrichment of DEGs in lipid metabolism-related pathways besides immune-related pathways indicated that lipid accumulation may also contribute to the immune response of Mφs encountering coaggregated *E. faecalis* and *F. nucleatum*. Therefore, we used Oil Red O staining to further validate the transcriptome analysis results and observe lipid accumulation in Mφs before and after infection.

After Oil Red O staining, lipids in Mφ were stained red, and nuclei were stained blue with hematoxylin. Microscopic observation showed varying degrees of lipid staining in all groups, with the coaggregated *E. faecalis* and *F. nucleatum* group having significantly more staining than the other groups, consistent with the quantitative detection results. Additionally, the *F. nucleatum* group had slightly more staining than the *E. faecalis* group (*p* < 0.05), and both had more than the negative control group (*p* < 0.01) (Figure 11B,C).

## 4. Discussion

Endodontic biofilm is an important factor of dental pulp infection, composed of various bacteria, among which *F. nucleatum* and *E. faecalis* are key participants. *F. nucleatum* has multiple adhesins and can act as a “bridge” to help with other bacteria attachment and form complex biofilms [21]. Furthermore, *F. nucleatum* may affect the ecosystem of biofilms and promote inflammatory responses through metabolic integration in dental pulp infections [22]. *E. faecalis* can form complex biofilms that adhere to the root canal wall and dentinal tubules and have high resistance to chemical and mechanical therapy (such as sodium hypochlorite irrigation) [23]. It was reported that *F. nucleatum* can bind to *E. faecalis* through the adhesion Fap2, promoting the integration of *E. faecalis* into the pre-established biofilm of *F. nucleatum* [24].

Under the stress of repeated root canal treatments, *F. nucleatum* and *E. faecalis* may form multicellular-like organisms, where the death of one subgroup provides better nutrient utilization for the overall survival and evolution of the microbial community, promoting the formation of community scaffolds and microbial diffusion channels. This allows the *F. nucleatum* and *E. faecalis* community to gain a stronger survival advantage as a whole, leading to persistent infections. Exploring the regulatory effects and mechanisms of their coaggregation on host immunity is of great significance for identifying treatment targets for refractory apical periodontitis.

Through TEM, we observed that after invasion of Mφs by coaggregated *E. faecalis* and *F. nucleatum*, both bacteria were located within the same phagosome, with extracellular matrix connections between them (Figure 1). This suggests that they are phagocytosed as a whole and synergistically regulate the host’s immune inflammatory and metabolic responses. Additionally, even after being phagocytosed, *E. faecalis* was still observed in a dividing state (Figure 1). It was first discovered in 2022 that *E. faecalis* could persist and proliferate within Mφs [25], which may provide opportunities for bacteria to evade immune clearance and spread within the body, potentially related to the persistence of host infections.

Furthermore, we found that Mφs infected with coaggregated *E. faecalis* and *F. nucleatum* or *F. nucleatum* alone showed significant nuclear shrinkage, while those infected with *E. faecalis* alone had relatively intact nuclear morphology (Figure 1). This suggests that Mφs infected with coaggregated *E. faecalis* and *F. nucleatum* or *F. nucleatum* may undergo programmed cell death accompanied by nuclear shrinkage. According to the literature, excessive ROS production and increased TNF-α production within cells can induce apoptosis and nuclear shrinkage [26,27], consistent with our previous findings that coaggregated *E. faecalis* and *F. nucleatum* or *F. nucleatum* infections could induce TNF-α production and promote Mφ apoptosis [15].

Interestingly, Mφs infected with coaggregated *E. faecalis* and *F. nucleatum* showed a significant increase in mitochondria. It has been reported that pathogens can promote mitochondrial proliferation in Mφs, thereby inducing the intake and usage of cellular lipid droplets and providing nutrients for pathogen survival and virulence factors synthesis [28,29,30,31].

Mφs can adapt their functions to different microenvironments by polarizing into different phenotypes, generally classified into two types: M1 (pro-inflammatory) and M2 (anti-inflammatory) [32]. In symptomatic apical periodontitis lesions, the proportion of M1/M2 Mφs significantly increases, suggesting that Mφ polarization plays a key role in the progression of apical periodontitis [5]. Our previous research found that coaggregated *E. faecalis* and *F. nucleatum* can promote the expression of pro-inflammatory cytokines IL-6 and TNF-α in Mφs, but the pro-inflammatory response is weaker compared to non-coaggregated bacteria, which may play a role in bacterial immune evasion and maintaining low-level cellular inflammation [15].

To further explore the effects of coaggregation of *E. faecalis* and *F. nucleatum* on Mφ, we used flow cytometry to detect Mφ polarization after infection with single-species and coaggregated bacteria. The results illustrated that cells in the *F. nucleatum* group and the coaggregated *E. faecalis* and *F. nucleatum* group showed significant M1 polarization 6 h after bacterial invasion, while the *E. faecalis* group only showed a small number of M1-polarized cells at 24 h (Figure 2).

According to the literature, *E. faecalis* can reduce the expression of pro-inflammatory cytokines in Mφs and inhibit M1 polarization, but its outer membrane vesicles (OMVs) can significantly induce M1 polarization [32]. Therefore, we speculate that the effect of *E. faecalis* on Mφ polarization may be related to the release amount and timing of its OMVs, but this needs further verification. Additionally, our experimental results indicate that coaggregated *E. faecalis* and *F. nucleatum* can promote M1 polarization of Mφ, which may be important for the formation of symptomatic apical periodontitis lesions.

Moreover, we found that coaggregated *E. faecalis* and *F. nucleatum* can induce ROS production in Mφ, with the amount produced being less than in the *E. faecalis* single-species group but similar to the *F. nucleatum* single-species group (Figure 3). This suggests that after coaggregation, *E. faecalis* and *F. nucleatum* may reduce ROS production in cells to favor bacterial survival, consistent with our previous finding that coaggregation increases the survival rate of *E. faecalis* within cells [15]. Meanwhile, coaggregated *E. faecalis* and *F. nucleatum* continuously induce low-level ROS production, which may sustain inflammation and promote the synergistic pathogenicity of the two pathogens, leading to the persistence of refractory apical periodontitis.

To further explore the mechanisms by which coaggregated *E. faecalis* and *F. nucleatum* affect Mφ immune inflammation and metabolic changes, we performed an analysis of RNA-seq. The results showed that coaggregated *E. faecalis* and *F. nucleatum* significantly altered the transcription levels of multiple genes in Mφ compared to single-species infections.

Interestingly, KDA analysis revealed that compared to *F. nucleatum* single-species infection, the 10 key driver genes regulating gene transcription-level changes in Mφs by coaggregated *E. faecalis* and *F. nucleatum* are all related to the chemokine signaling pathway, with most showing significant upregulation (Figure 10). Among them, the upregulated *CXCL1*, *CXCL2*, and *CXCL3* attract neutrophils through the same receptor CXCR2.

Studies have shown that in chronic inflammation, Mφ recruitment of neutrophils may exacerbate the persistence of inflammation. Neutrophils may release pro-inflammatory cytokines, activate Mφ, and recruit more immune cells, forming a vicious cycle that leads to sustained Mφ activation and production of pro-inflammatory mediators, thereby maintaining the inflammatory environment [33]. The above results suggest that *E. faecalis* may assist *F. nucleatum* in inducing Mφ secretion of chemokines and expression of chemokine receptors, thereby causing a series of changes in cellular immune metabolism and inflammatory responses, aggravating refractory apical periodontitis.

Furthermore, compared with *E. faecalis* single-species infection, the apoptosis-related gene *Fas* in Mφs was upregulated by 3.6-fold after coaggregated *E. faecalis* and *F. nucleatum* infection, which was subsequently verified by qRT-PCR (Figure 11A). This result may explain the phenomenon of significant nuclear shrinkage of Mφs infected with coaggregated *E. faecalis* and *F. nucleatum* (Figure 1). It was reported that excessive apoptosis may promote the release of intracellular bacteria, thereby facilitating the spread of infection and aggravating tissue damage [3].

Interestingly, besides genes related to inflammatory responses, coaggregated *E. faecalis* and *F. nucleatum* significantly upregulated the expression of genes related to lipid metabolism pathways compared to both *E. faecalis* and *F. nucleatum* single-species infections (Figure 5C and Figure 8C). This suggests that Mφ lipid metabolism may also be involved in the immune regulation by coaggregated *E. faecalis* and *F. nucleatum*. Our study found that coaggregation of *E. faecalis* and *F. nucleatum* upregulated the expression of the lipid metabolism-related gene *Steroid Receptor Coactivator* (*SRC*) by approximately 18-fold compared to *E. faecalis* alone (Table S2).

SRC is a tyrosine kinase closely related to host lipid metabolism and is located downstream of the Toll-like receptor 9 (TLR9) signaling pathway. Upon recognition of bacterial DNA by TLR9, SRC can activate the host immune response [34,35]. SRC can promote the phosphorylation of TANK binding kinase 1 (TBK1), thereby facilitating the production of type I interferon (IFN-I) [36,37]. IFN-I can promote Mφ to uptake cholesterol through scavenger receptor-A1 (SR-A1), inducing intracellular lipid droplet formation and lipid accumulation and leading to foam cell formation [38].

Indeed, through KDA analysis, we found that the three key driver genes significantly upregulated by coaggregated *E. faecalis* and *F. nucleatum* compared to *E. faecalis* are all IFN-stimulated genes. Among them, *RSAD2*, with the most node connections, can induce lipid droplet formation and lipid synthesis [39], suggesting that the lipid- and atherosclerosis-related pathways may not only be involved in Mφ inflammatory responses but also serve as a key hub for regulating inflammation.

Next, we examined the effect of coaggregation of *E. faecalis* and *F. nucleatum* on lipid accumulation in Mφ. The experiment found varying degrees of lipid staining in all groups, with the coaggregated *E. faecalis* and *F. nucleatum* group showing significantly more staining than the other groups (Figure 11B).

Lipid accumulation in Mφ plays an important role in the survival and persistent infection of intracellular bacteria. While providing nutrients for bacteria, it can interfere with phagosome acidification, creating a neutral pH environment to support bacterial survival [28,30]. Additionally, bacteria can utilize lipid droplets to generate dodecanoic acid-like substances to regulate inflammatory responses and evade immune system clearance [40]. Importantly, lipid accumulation can also trigger mitochondrial production of ROS, activating the NLRP3 inflammasome and inducing the production of pro-inflammatory cytokines such as IL-6, IL-1β, IL-18, and TNF-α, forming a positive feedback loop to maintain chronic inflammation [41].

Our previous research found that after coaggregation of *E. faecalis* and *F. nucleatum*, the expression levels of a series of genes changed, with the *cidA* gene in *F. nucleatum* upregulated by 9.9-fold [15], and we used qRT-PCR to verify the result (Figure S4). CidA is an autolysin, a complete membrane protein that can form large protein aggregates on the cytoplasmic membrane through oligomerization, composing transmembrane pores that depolarize the membrane and activate peptidoglycan hydrolases and triggering bacterial programmed cell death (PCD) [42,43].

PCD refers to the genetically encoded process by which bacteria actively initiate cell death under specific conditions, an important mechanism for bacterial communities to cope with environmental stress and maintain dynamic balance [44]. Under environmental stress, some bacteria reduce population density through PCD while releasing macromolecules such as DNA, RNA, and proteins as nutrients for other bacteria to maintain community survival [45].

Bacteria release extracellular DNA (eDNA) through PCD, which is a major source of eDNA. eDNA can cross-link with proteins in the bacterial extracellular matrix, promoting adhesion between bacteria and the stability of biofilm spatial structures [46,47,48]. Notably, abnormal DNA can promote cholesterol uptake by Mφs through scavenger receptors, leading to intracellular lipid droplet formation and lipid accumulation, forming foam cells [38].

Therefore, we speculate that *F. nucleatum* may induce lipid accumulation in Mφs by releasing eDNA after PCD induced by upregulated CidA, thereby assisting *E. faecalis* in surviving within Mφs, which needs further verification.

In this study, we used various methods to investigate the regulatory effects and mechanisms of *E. faecalis* and *F. nucleatum* coaggregation on Mφ ultrastructural, polarized, and metabolic changes. We found that coaggregated *E. faecalis* and *F. nucleatum* could cause nuclear shrinkage and increased mitochondria in Mφ, while inducing M1 polarization, ROS production, and lipid accumulation of Mφ. Also, the coaggregated bacteria could change the transcription levels of a series of genes in Mφ.

Such analysis could be extended to later time points using advanced sequencing technology (such as dual RNA-seq), and this needs corresponding result verification, which may hold considerable value as a new perspective on the treatment targets of refractory apical periodontitis.

## Data Availability

The raw data of the RNA sequencing supporting the findings of this study have been deposited in the NCBI Sequence Read Archive (SRA) under accession number PRJNA1243572.

## Supplementary Materials

The following supporting information can be downloaded at https://www.mdpi.com/article/10.3390/microorganisms13061351/s1. Figure S1: Heatmap of the differentially expressed genes (DEGs) in different groups; Figure S2: Protein–protein interaction (PPI) network analysis of DEGs of coaggregated *E. faecalis* and *F. nucleatum* group vs. *E. faecalis* group; Figure S3: PPI network analysis of DEGs of coaggregated *E. faecalis* and *F. nucleatum* group vs. *F. nucleatum* group; Figure S4: qRT-PCR validation of expression of *cidA* in *F. nucleatum* before and after coaggregation; Table S1: Sequences of qRT-PCR primers; Table S2: DEGs in Mφs infected with coaggregated *E. faecalis* and *F. nucleatum* vs. *E. faecalis*; Table S3: DEGs in Mφs infected with coaggregated *E. faecalis* and *F. nucleatum* vs. *F. nucleatum*.
