# Supplementary material for: Interaction Between Enterococcus faecalis and Fusobacterium nucleatum Regulated Macrophage Transcriptional Profiling and Reprogrammed Cellular Immune and Metabolic Response
Source: Microorganisms. 2025 Jun 11;13(6):1351. doi: 10.3390/microorganisms13061351 (PMC12195608; doi:10.3390/microorganisms13061351)
Supplement: Supplementary file 1 [file microorganisms-13-01351-s001.zip › Table S1 Sequences of qRT-PCR Primers.pdf]

Table S1. Sequences of qRT-PCR Primers

Genes		Sequence
SRC	Forward	5′-TGGCAAGATCACCAGACGG-3′
	Reverse	5′-GGCACCTTTCGTGGTCTCAC-3′
OAS1	Forward	5′-TGTCCAAGGTGGTAAAGGGTG-3′
	Reverse	5′-CCGGCGATTTAACTGATCCTG-3′
GBP1	Forward	5′-AGGAGTTCCTTCAAAGATGTGGA-3′
	Reverse	5′-GCAACTGGACCCTGTCGTT-3′
RSAD2	Forward	5′-TTGGACATTCTCGCTATCTCCT-3′
	Reverse	5′-AGTGCTTGTCTTTCCGTC-3′
Fas	Forward	5′-TCTGGTTCTTACGTCTGTTGC
	Reverse	5′-CTGTGCAGTCCCTAGCTTTCC-3′
CXCL1	Forward	5′-GCCCAAACCGAAGTCATAGCC-3′
	Reverse	5′-ATCCGCCAGCCTCTATCACA-3′
CXCL2	Forward	5′-CTCAAGAACATCCAAAGTGTG-3′
	Reverse	5′-ATTCTTGAGTGTGGCTATGAC-3′
CXCL3	Forward	5′-CCAAACCGAAGTCATAGCCAC-3′
	Reverse	5′-GCTCCCCTTGTTCAGTATCT-3′
GAPDH	Forward	5′-ACAACTTTGGTATCGTGGAAGG-3′
	Reverse	5′-GCCATCACGCCACAGTTTC-3′
