# Supplementary figures and images for: Interaction Between Enterococcus faecalis and Fusobacterium nucleatum Regulated Macrophage Transcriptional Profiling and Reprogrammed Cellular Immune and Metabolic Response
Source: Microorganisms. 2025 Jun 11;13(6):1351. doi: 10.3390/microorganisms13061351 (PMC12195608; doi:10.3390/microorganisms13061351)

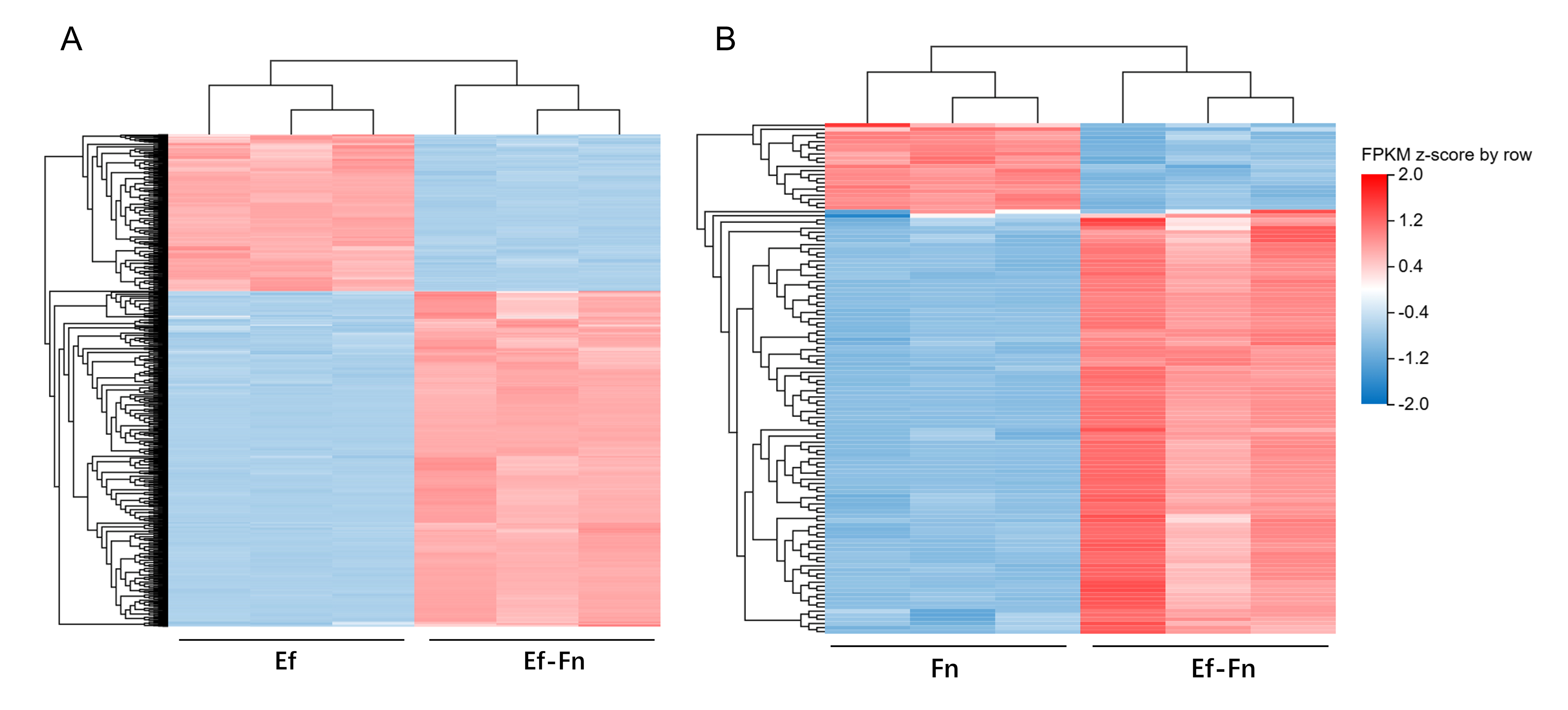

Supplement: Supplementary file 1 [file microorganisms-13-01351-s001.zip › Figure S1 Heatmap of the DEGs in different groups. Squares in red and blue represent upregulated and downregulated genes, respectively..tif]

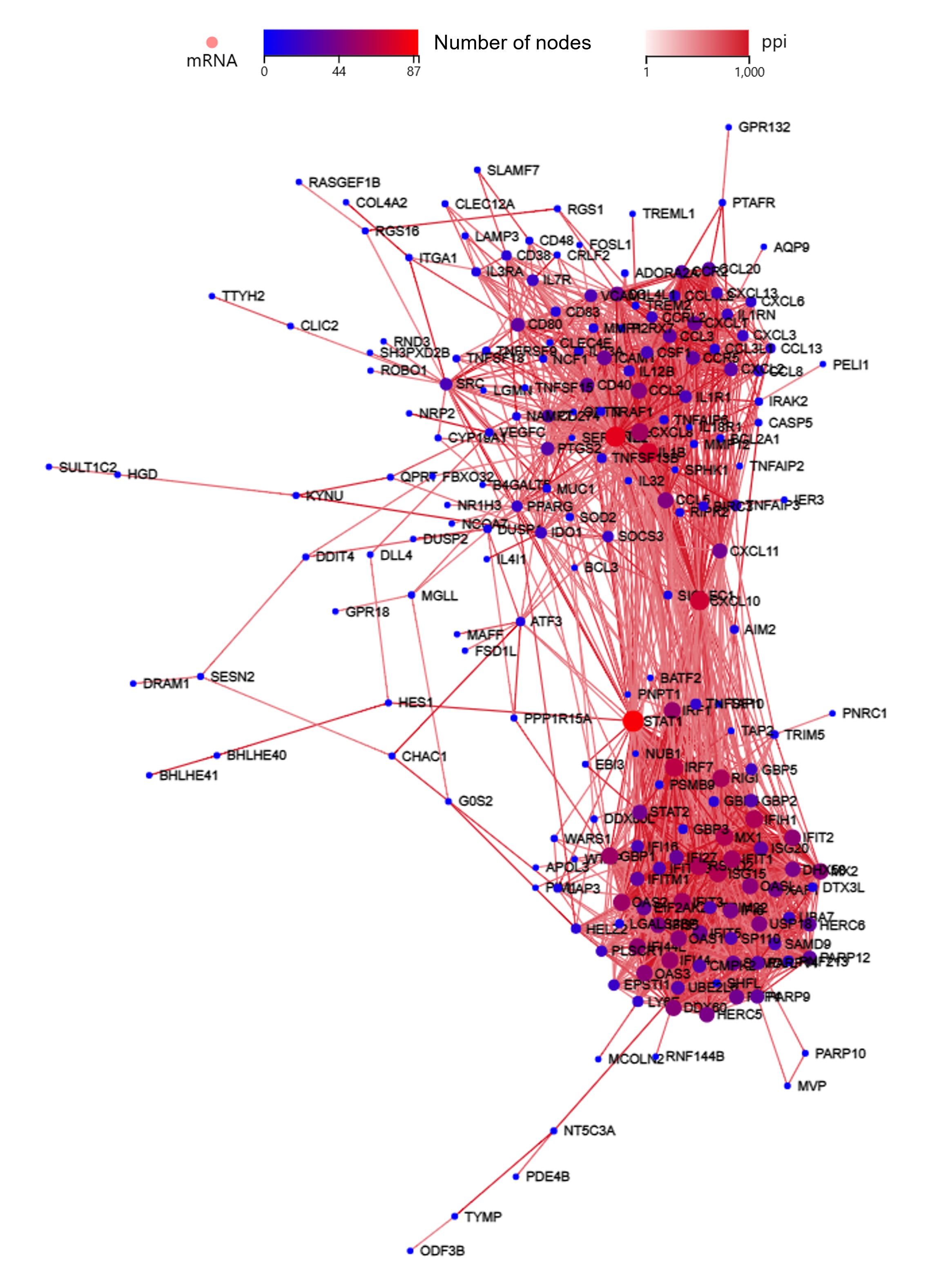

Supplement: Supplementary file 1 [file microorganisms-13-01351-s001.zip › Figure S2 PPI network analysis of DEGs of Coaggregated E. faecalis and F. nucleatum VS E. faecalis.tif]

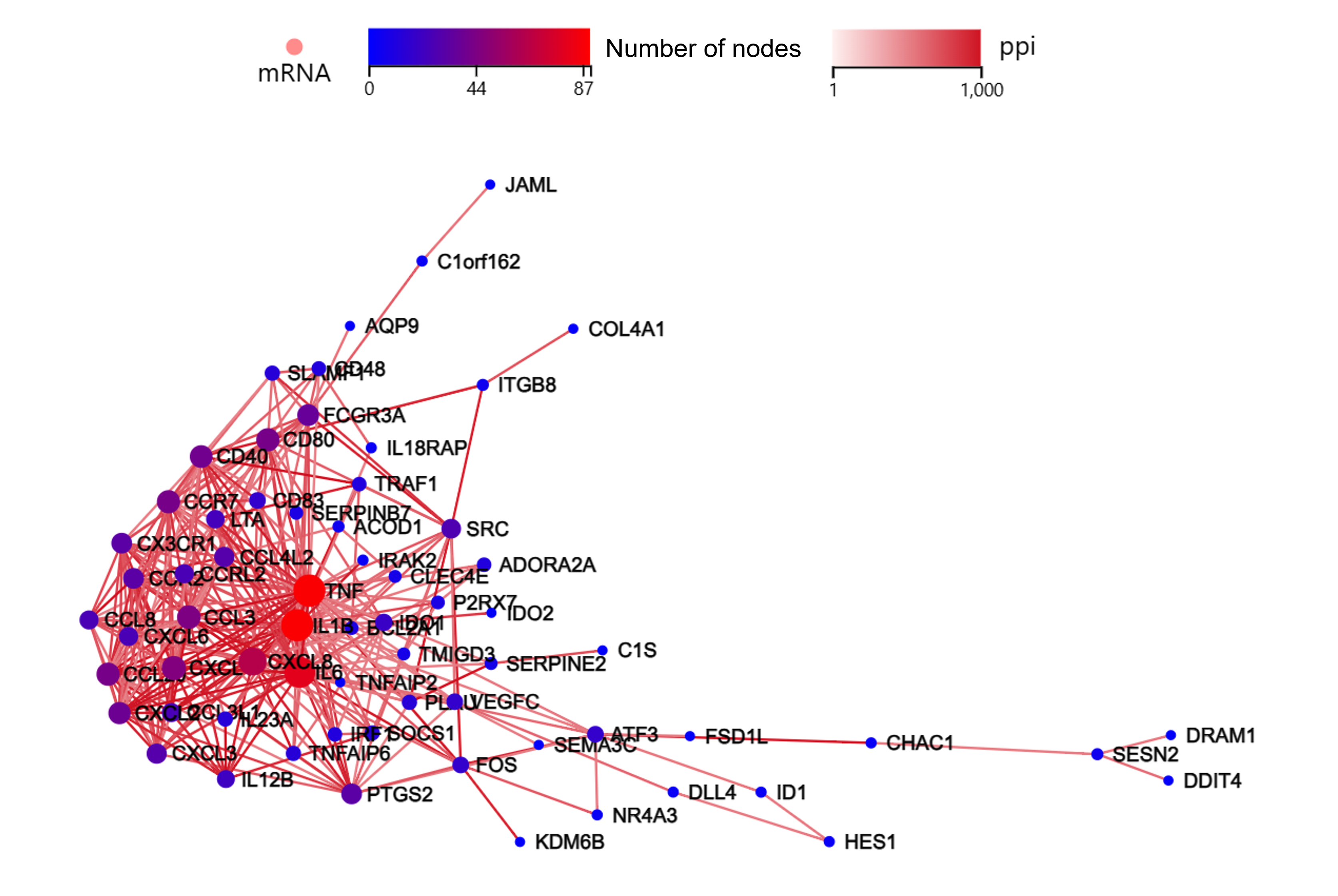

Supplement: Supplementary file 1 [file microorganisms-13-01351-s001.zip › Figure S3 PPI network analysis of DEGs of Coaggregated E. faecalis and F. nucleatum VS F. nucleatum.tif]

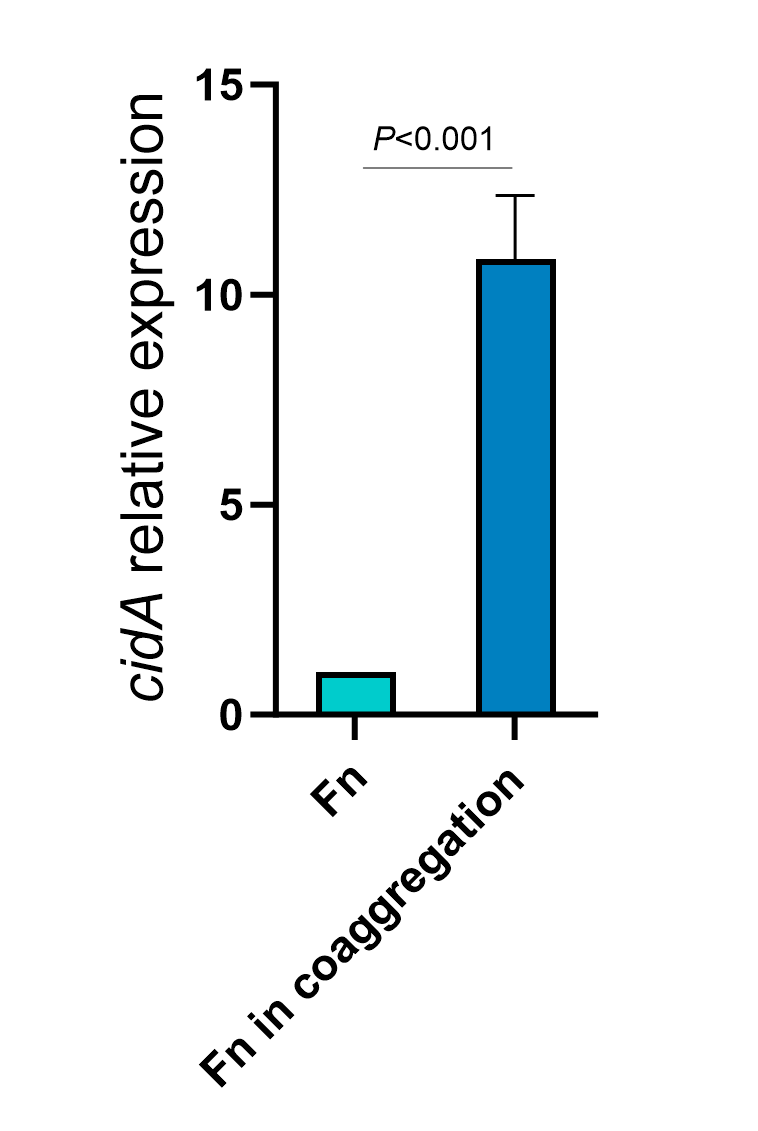

Supplement: Supplementary file 1 [file microorganisms-13-01351-s001.zip › Figure S4 qRT-PCR Validation of Expression of cidA in F. nucleatum before and after coaggregation..tif]
